# Supplementary material for: Large optical nonlinearity of ITO nanorods for sub-picosecond all-optical modulation of the full-visible spectrum
Source: Nat Commun. 2016 Sep 29;7:12892. doi: 10.1038/ncomms12892 (PMC5056417; doi:10.1038/ncomms12892)
Supplement: Supplementary Information — Supplementary Figures 1-16, Supplementary Notes 1-8 and Supplementary References [file ncomms12892-s1.pdf]

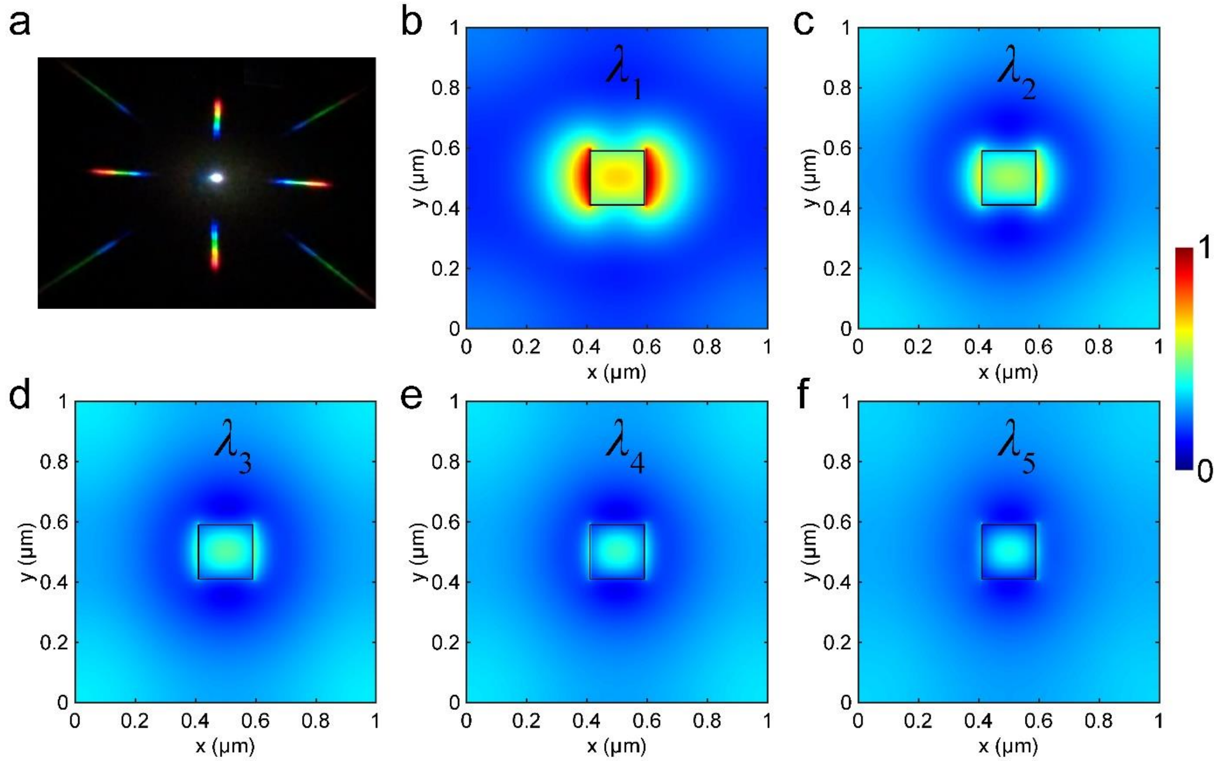

Supplementary Figure 1 | Higher order diffraction and electric field distribution at the transmission minima. a, Photograph of the zero and higher grating orders produced by diffraction of a broadband visible probe by the ITO-NRA sample at normal incidence. **b** to **f**, Simulated total electric field distributions at transmission minima λ_1 to λ_5, averaged over the length of the nanorod.

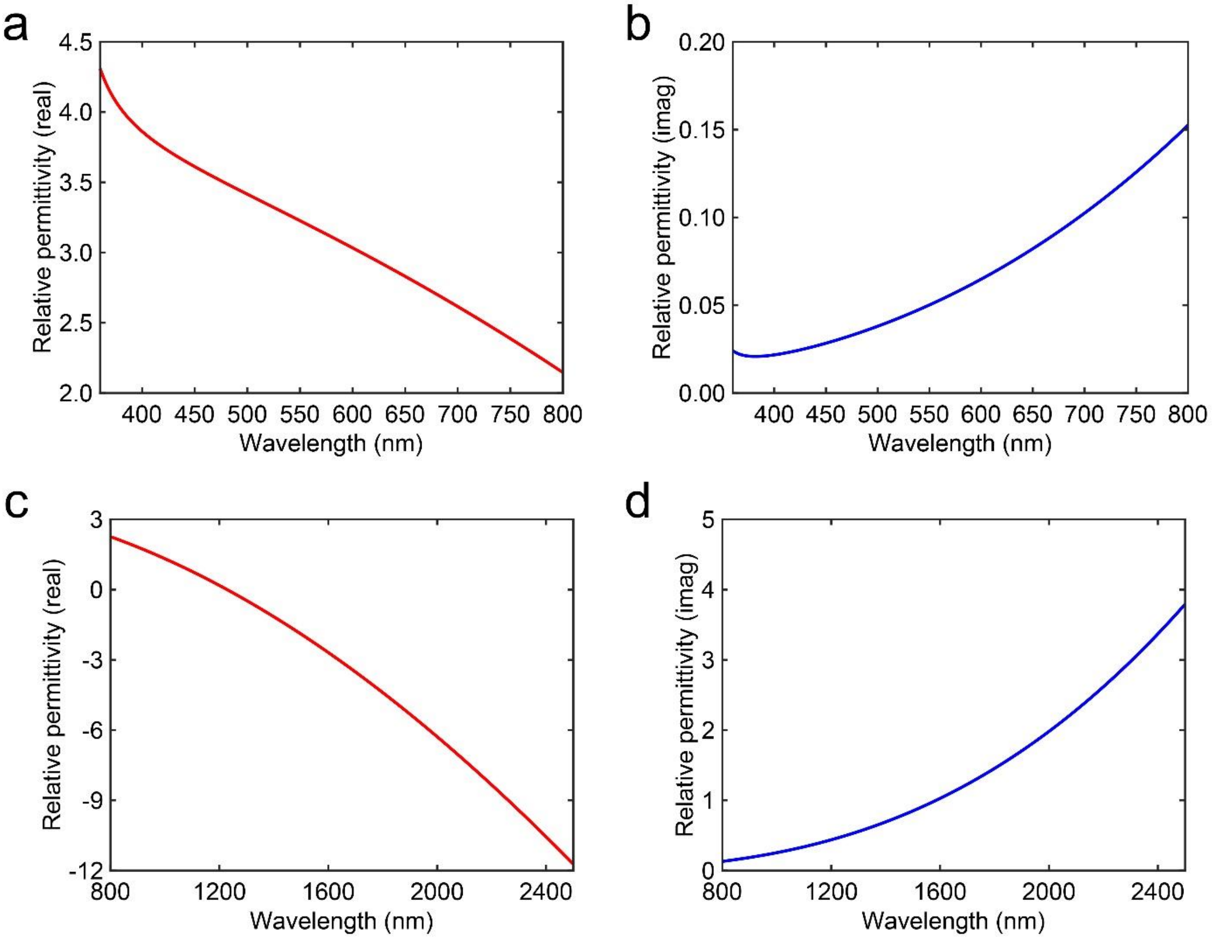

Supplementary Figure 2 | Permittivity of ITO in the visible and near-infrared range. a and **b**, Real and imaginary parts of the relative permittivity in the visible range (using the Drude-Lorentz model). **c** and **d**, Real and imaginary parts of the relative permittivity in the near-infrared range (using the Drude model).

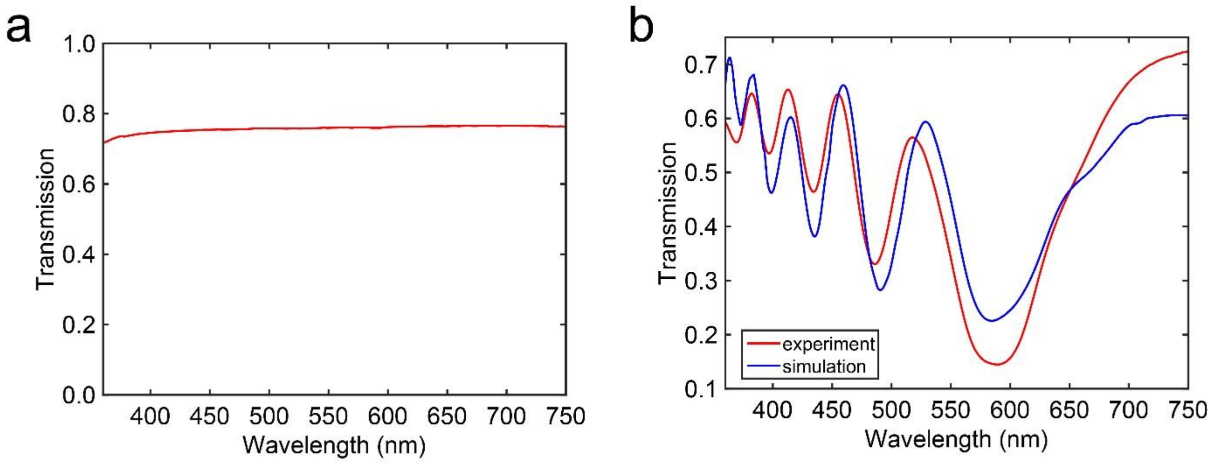

Supplementary Figure 3 | Substrate transmission and comparison of simulated and experimental transmission spectra of the ITO-NRA. a, Transmission spectrum of a 0.5 mm thick YSZ substrate. **b**, Comparison of the simulated and experimental spectra of the array. The ITO permittivity used in the optical simulation is shown in Supplementary Fig. 2a and Fig. 2b.

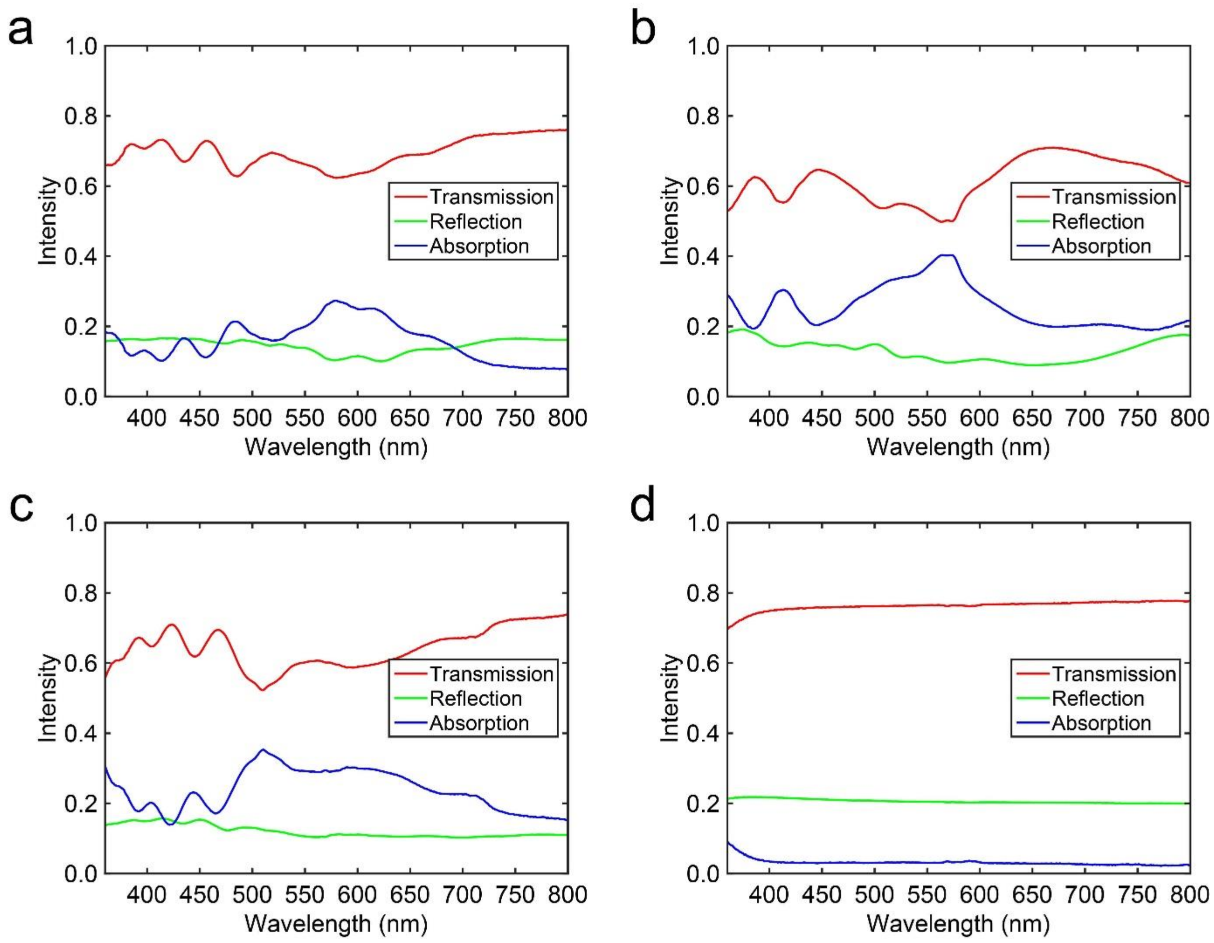

Supplementary Figure 4 | Static optical measurements using an integrating sphere. a, **b** and **c**, Total transmission, total reflection and deduced absorption spectra for the ITO-NRAs. The periodicities of ITO-NRAs in **a**, **b** and **c** are 1 µm, 800 nm and 1 µm, respectively, and the heights of ITO-NRAs are 2.6 µm, 1.4 µm and 2.9 µm, respectively. **d**, Total transmission, total reflection and deduced absorption spectra for a bare YSZ substrate (0.5 mm thick).

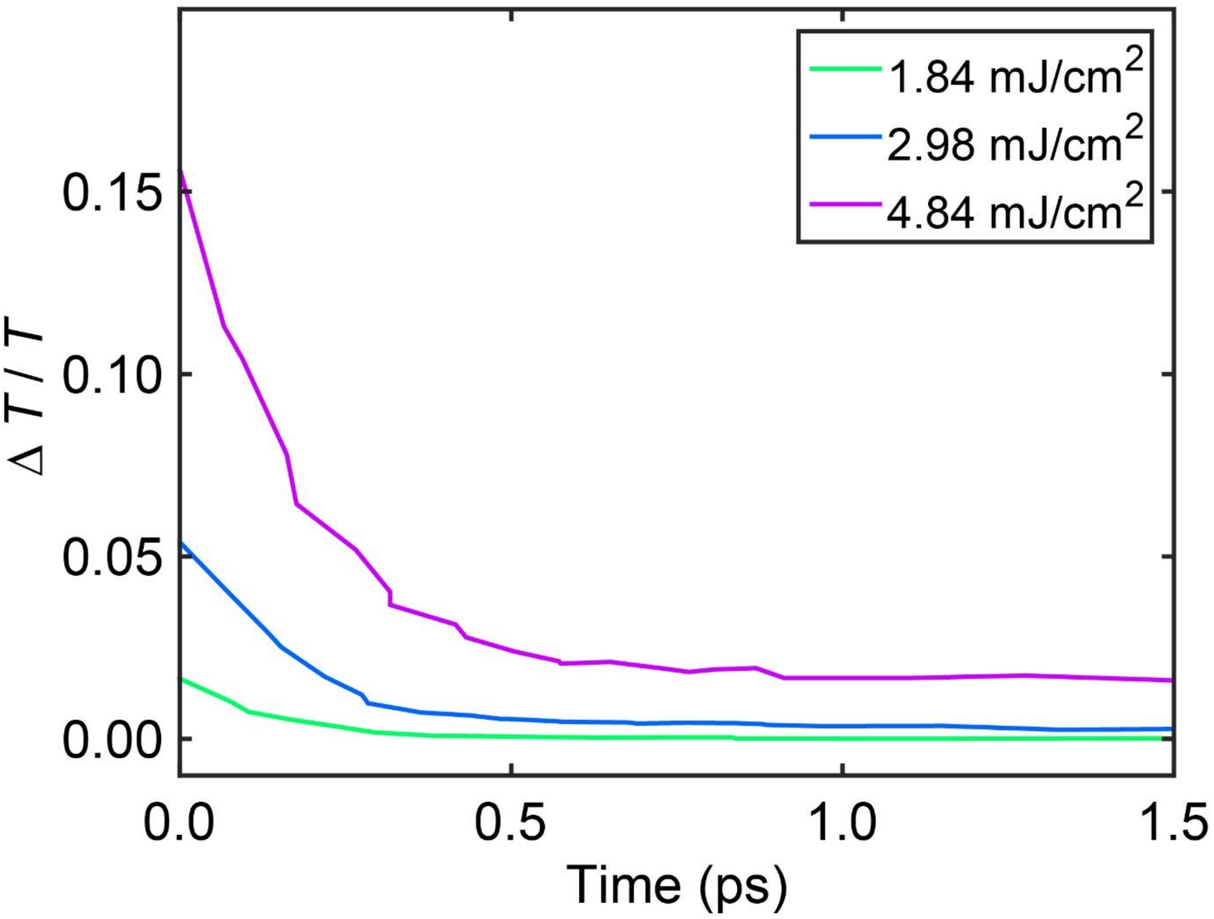

Supplementary Figure 5 | Off-resonance pumping of the ITO-NRA. $\Delta T(t)/T(0)$ kinetics of the ITO-NRA at 568 nm. The sample is pumped at 800 nm.

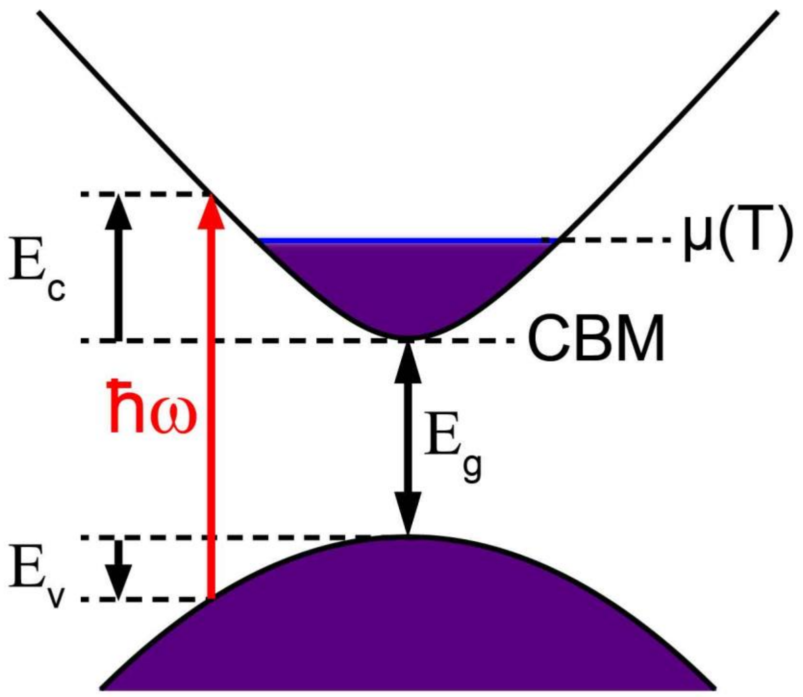

Supplementary Figure 6 | Schematic diagram of the direct interband optical transition in a semiconductor with a non-parabolic conduction band and a parabolic valence band.

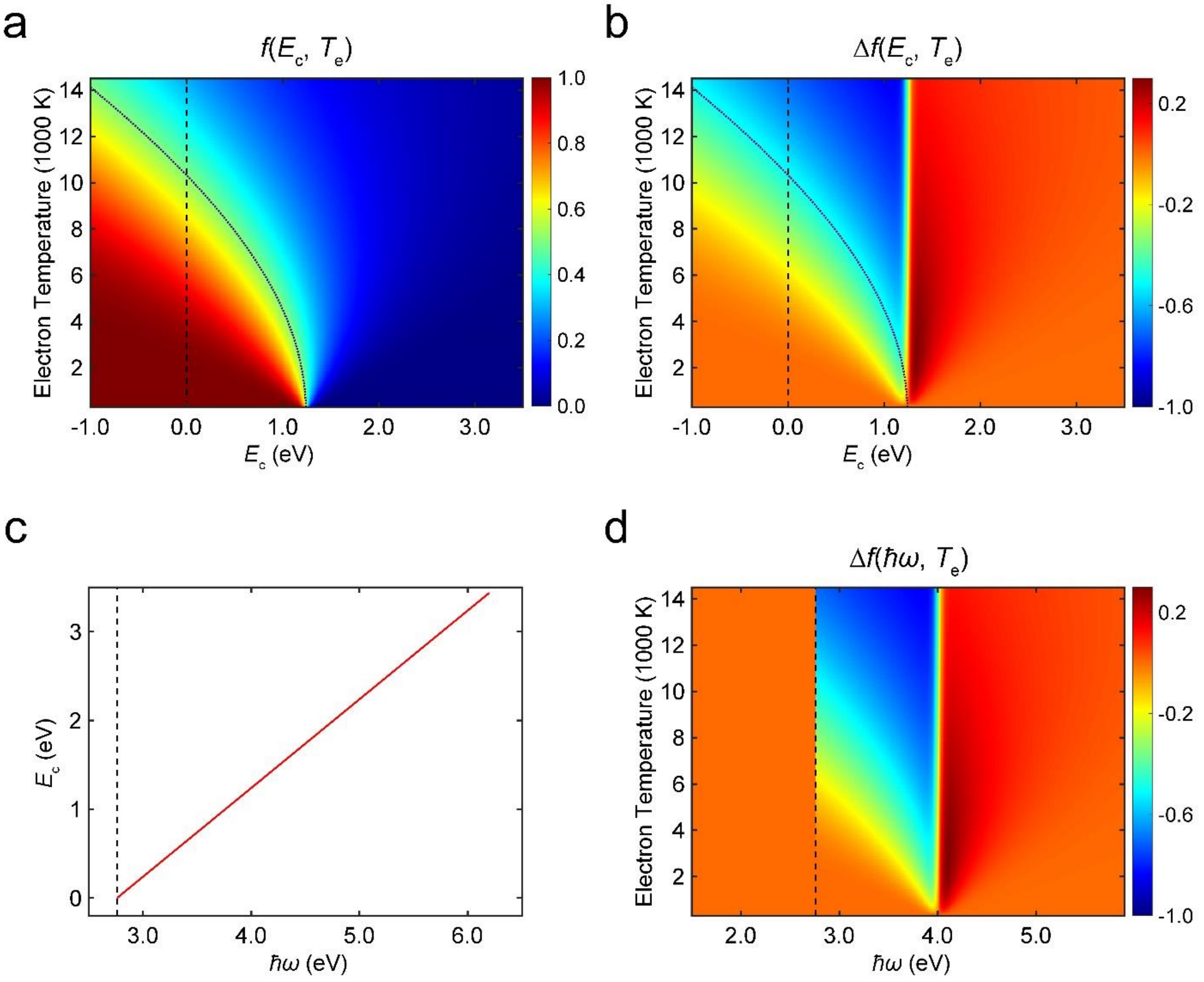

Supplementary Figure 7 | Theoretical calculation of the change of electron distribution. a, Electron distribution as a function of E_c and electron temperature. **b**, Change of electron distribution as a function of E_c and electron temperature. **c**, E_c as a function of photon energy $\hbar\omega$. **d**, Change of electron distribution as a function of photon energy $\hbar\omega$ and electron temperature. The vertical dashed line in **a**, **b**, **c** and **d** indicates the conduction band minimum. The dotted line in **a** and **b** indicates the temperature dependent electron chemical potential μ (or equivalently the Fermi energy).

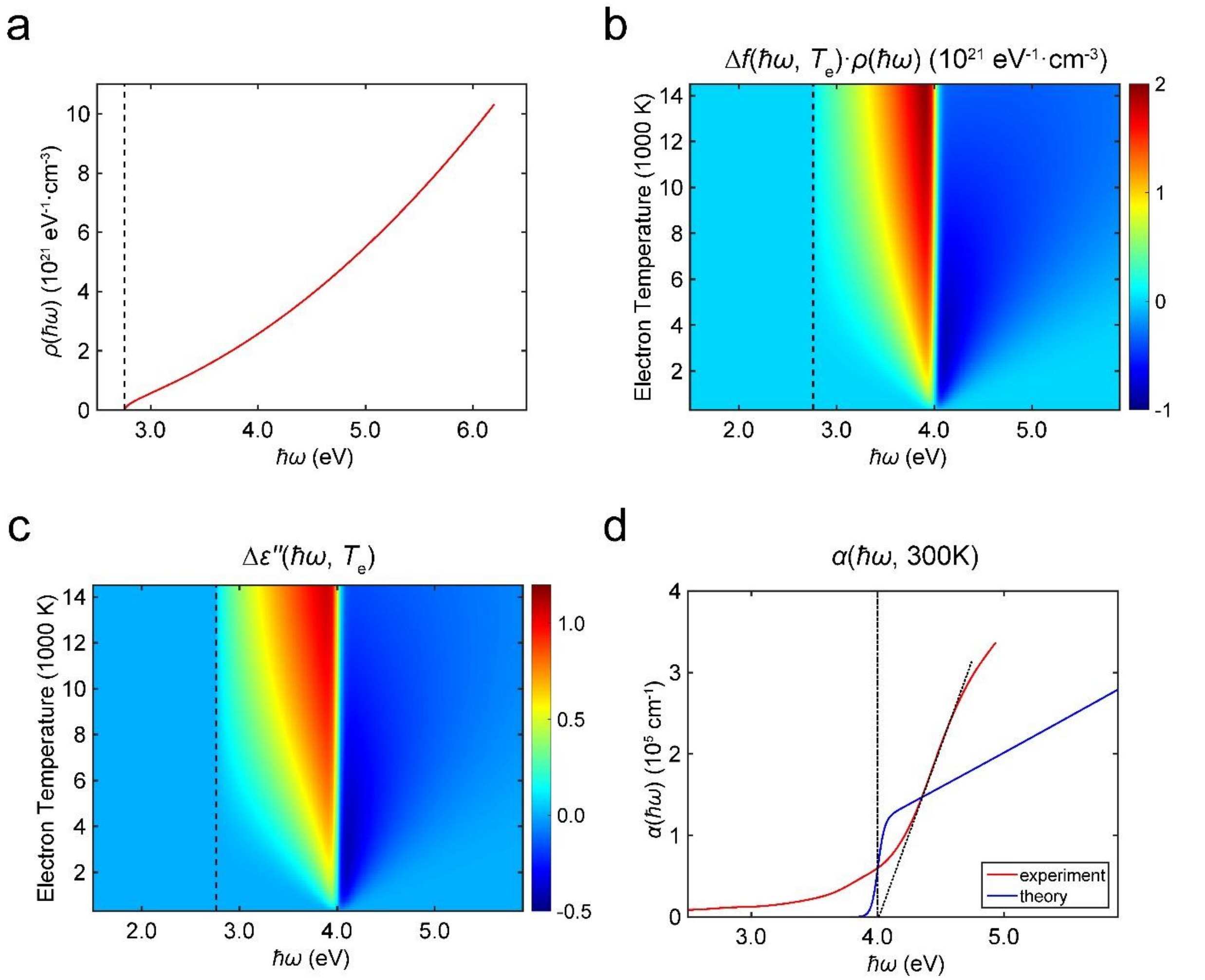

Supplementary Figure 8 | Theoretical calculation of the change of imaginary part of permittivity. **a**, Joint-density-of-states $\rho(\hbar\omega)$ as a function of photon energy $\hbar\omega$. **b**, Product of the electron distribution change and $\rho(\hbar\omega)$ as a function of photon energy and electron temperature. **c**, Change of the imaginary part of the relative permittivity as a function of photon energy and electron temperature. The vertical dashed line in **a**, **b**, **c** indicates the conduction band minimum. **d**, Red line: $\alpha(\omega)$ obtained from ellipsometry measurement of an ITO film. Blue line: theoretically calculated $\alpha(\omega)$ for the case of a constant matrix element of $(2.8\times10^{-49}\ \text{J}\cdot\text{kg})^{1/2}$. The dash-dot line in **d** represents the absorption onset energy, $E_g + \mu(300\ \text{K})$, obtained by extrapolating the straight dotted line to the $\alpha = 0$ axis.

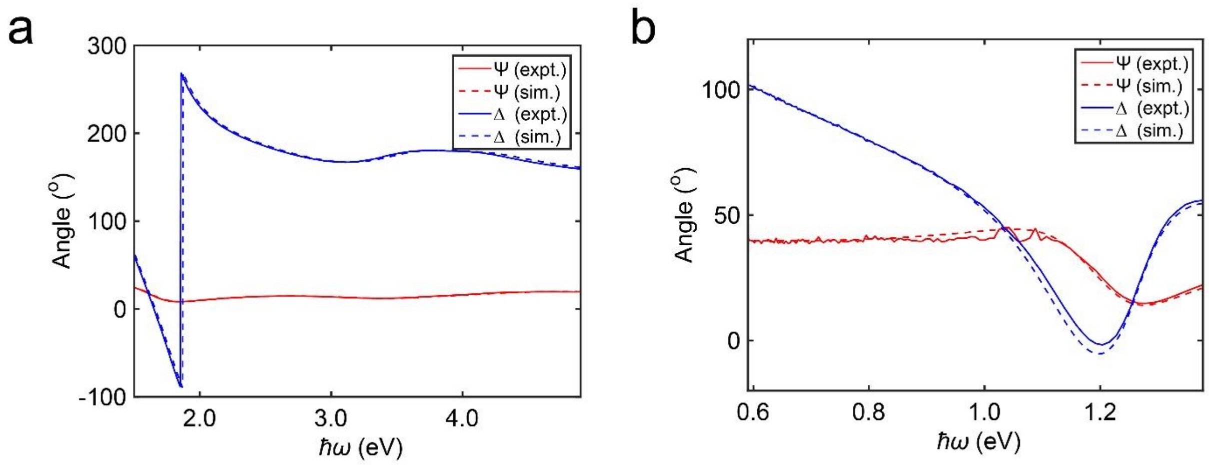

Supplementary Figure 9 | Ellipsometric measurements of ITO film. a, Experimental and simulated Ψ and Δ angles for the ITO film in the ultraviolet to the visible range; the corresponding refractive indices from the ellipsometric data fitting were used to calculate $\alpha(\omega)$ shown in Supplementary Fig. 8d. The experimental data was acquired at 55° using a J. A. Woollam M2000U instrument. **b**, Experimental and simulated Ψ and Δ angles for the ITO film in the NIR range; the data fitting assumed a Drude permittivity with ε_∞ = 3.90, ω_p = 2.10 eV, and γ_p = 0.0.065 eV. The experimental data was acquired at 70° using a Horiba UVISEL instrument.

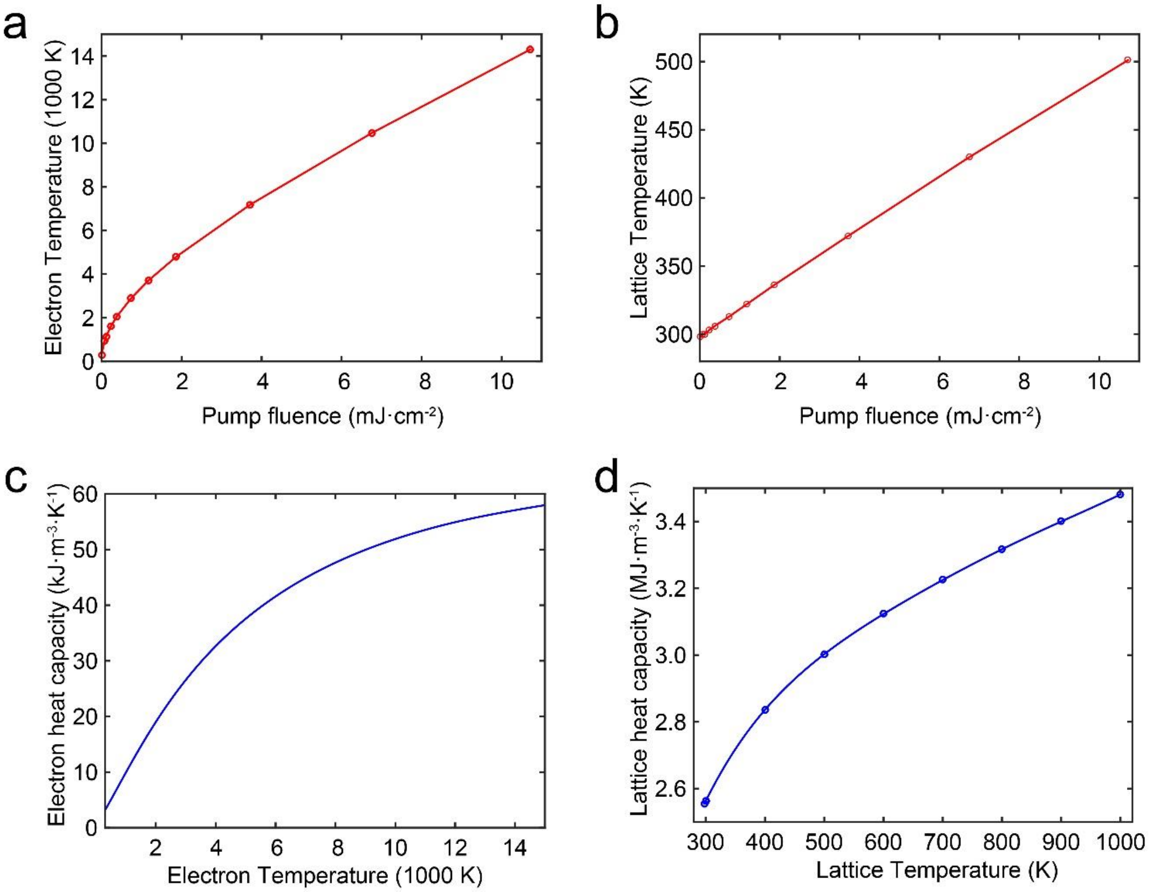

Supplementary Figure 10 | Heat capacities and initial temperatures of the electron and lattice. **a**, Electron temperature $T_{e,0}$ as a function of pump fluence. **b**, Lattice temperature $T_{l,0}$ as a function of pump fluence. In both **a** and **b**, the curves are numerically calculated results, whereas the circles are associated with actual pump fluences used in the short-delay-TA experiments. **c**, Temperature dependent heat capacity of the electron gas. **d**, Temperature dependent heat capacity of the lattice (circles: measured data points[1]; curve: interpolated dependence).

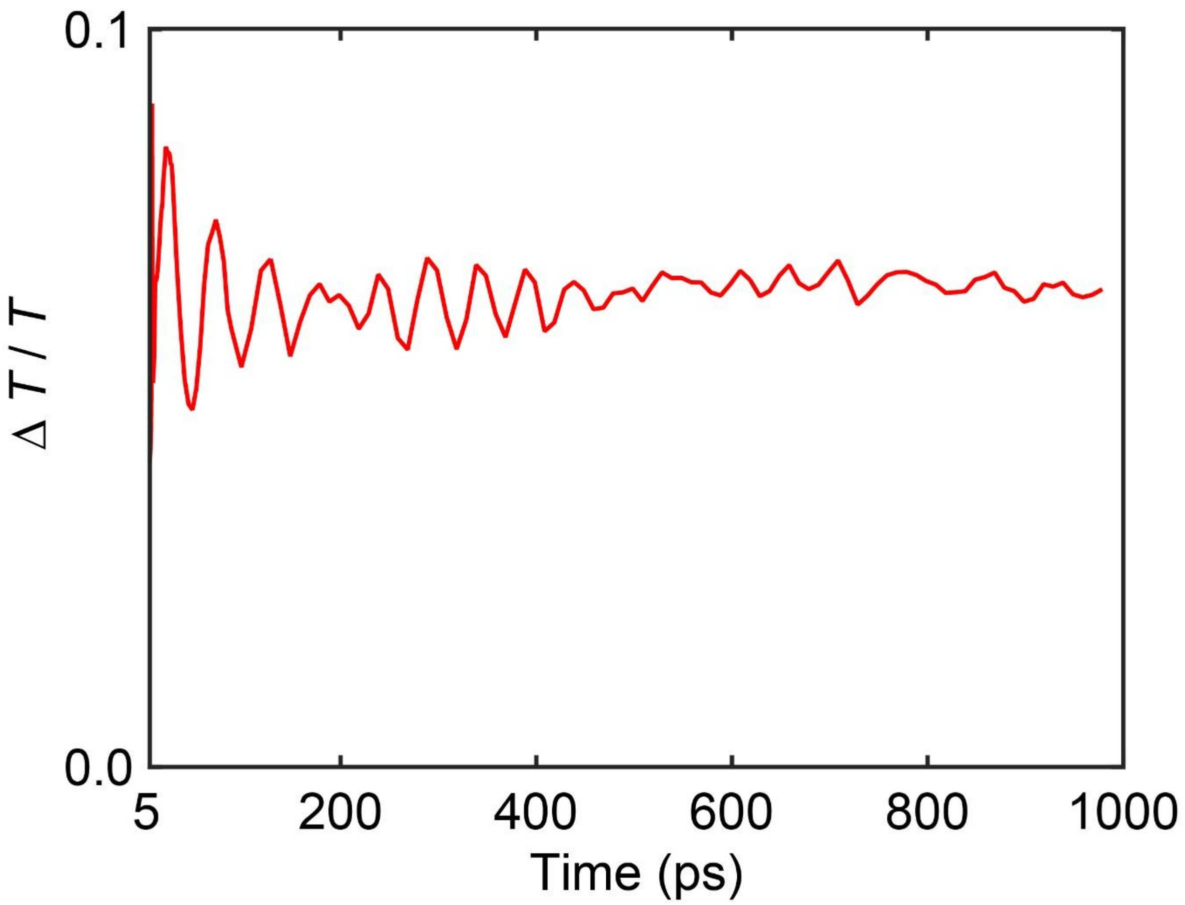

Supplementary Figure 11 | Dephasing of the acoustic vibrations of the ITO-NRA. $\Delta T(t)/T(0)$ spectrum of the ITO-NRA at 475 nm, arising due to the excitation of acoustic vibrations.

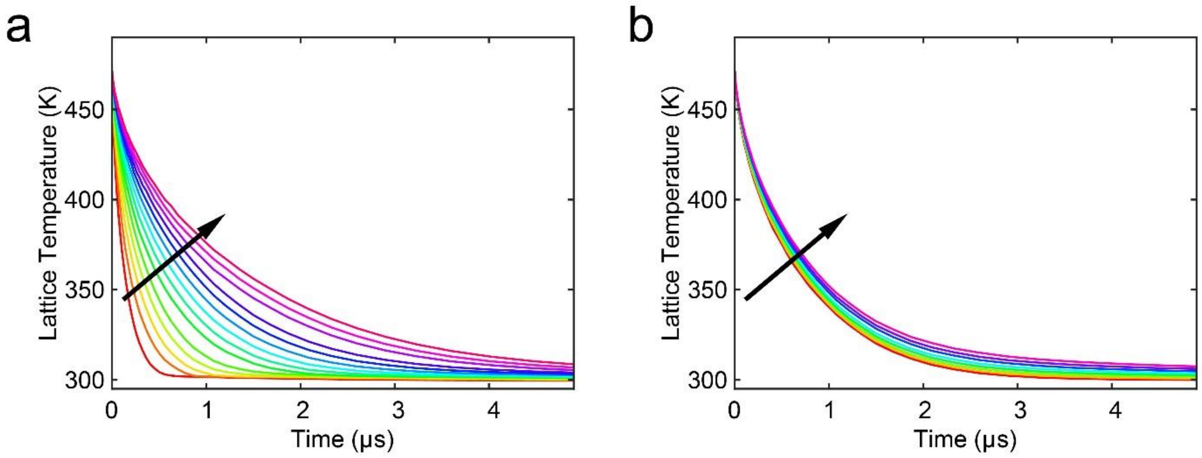

Supplementary Figure 12 | Heat-transfer simulation results of the temperature decay of the lattice. Temporal decay of $(\int T_\mathrm{L}\mathrm{d}V)/V$ (which is lattice temperature averaged over the nanorod volume) with a uniform initial temperature of 474 ºC. **a**, Edge length is fixed at 180 nm. Arrow indicates increasing the nanorod height from 1 μm to 3.6 μm with a step size of 200 nm. **b**, Height is fixed at 2600 nm. Arrow indicates increasing the edge length from 120 nm to 260 nm with a step size of 20 nm. Periodicity of the ITO-NRA in all heat-transfer simulations is 1 μm.

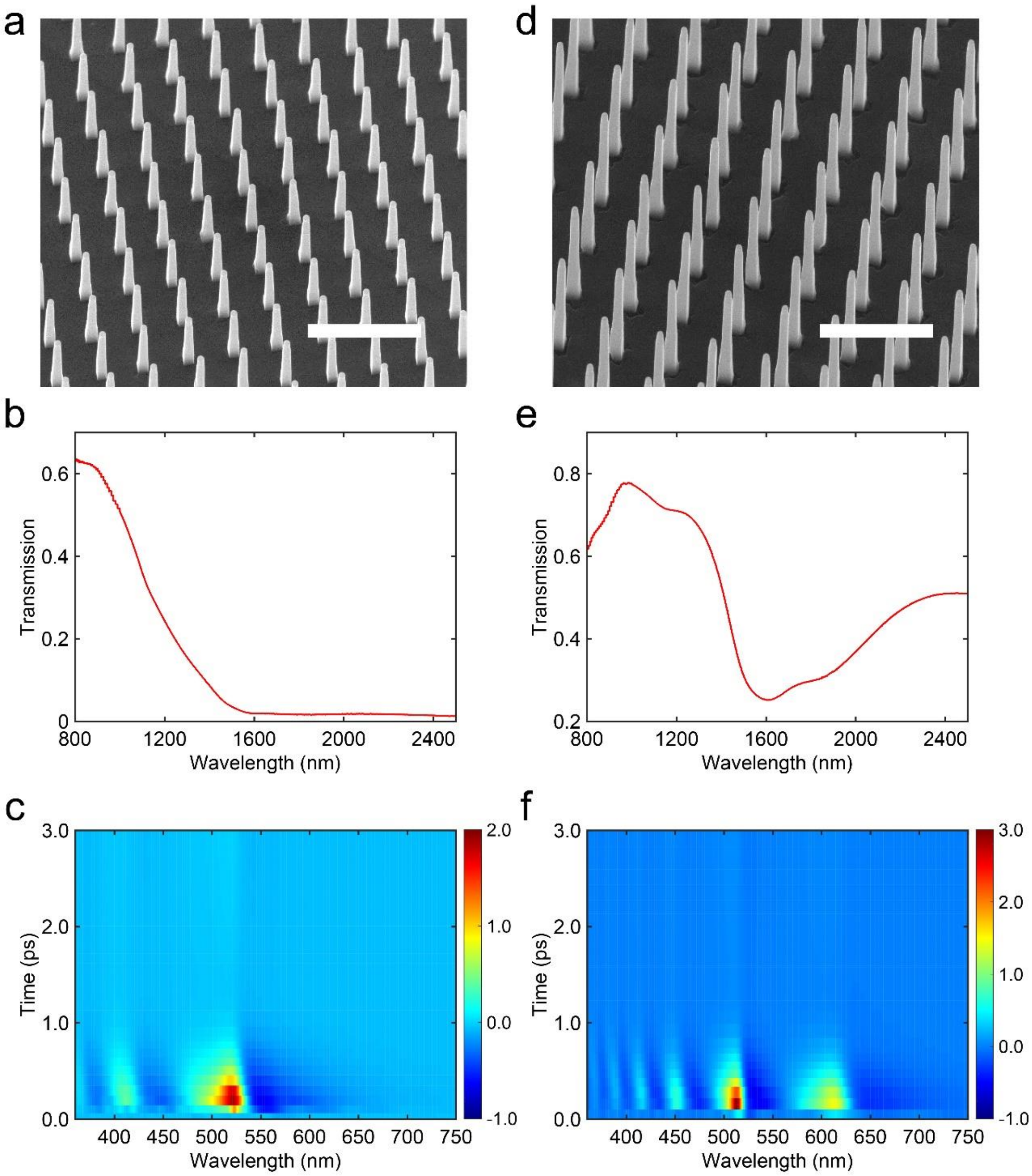

Supplementary Figure 13 | Morphologies and optical properties of additional ITO-NRA samples. a, **b**, **c**, SEM image, static near-infrared transmission spectrum and $\Delta T(t)/T(0)$ spectral map for an ITO-NRA with 1.4 μm height. Scale bars in **a** and **b** are 2 μm. **d**, **e**, **f**, SEM image, static near-infrared transmission spectrum and $\Delta T(t)/T(0)$ spectral map for an ITO-NRA with 2.9 μm height. Pump fluence in **c** and **f** is 7.4 mJ·cm^{-2}.

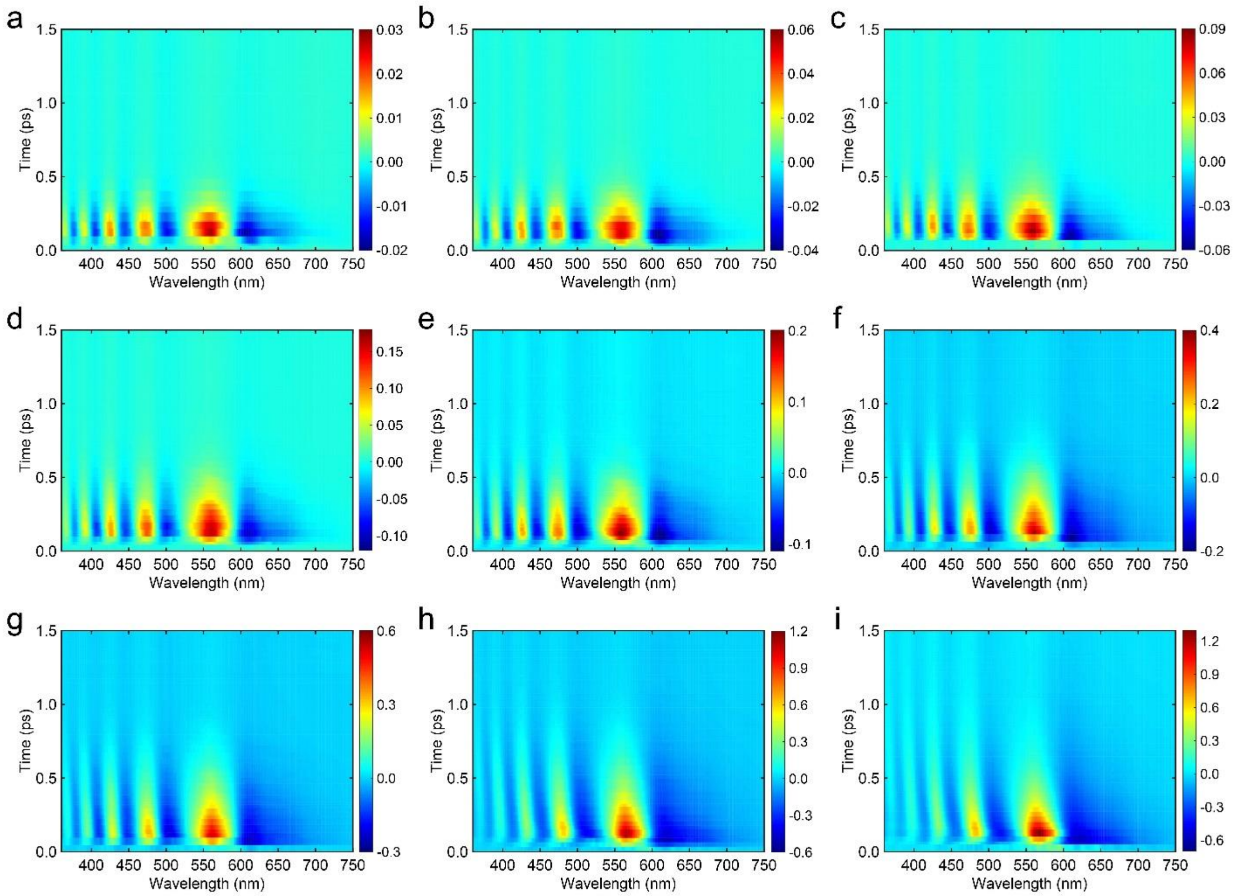

Supplementary Figure 14 | Transient spectral maps of the fast component. $\Delta T(t)/T(0)$ spectral maps for the ITO-NRA plotted for delay times up to 1.5 ps (acquired from the short-delay-TA experiments). Pump fluences for **a** to **i** are 0.073 mJ·cm^{-2}, 0.114 mJ·cm^{-2}, 0.235 mJ·cm^{-2}, 0.373 mJ·cm^{-2}, 0.739 mJ·cm^{-2}, 1.177 mJ·cm^{-2}, 1.859 mJ·cm^{-2}, 6.755 mJ·cm^{-2} and 10.72 mJ·cm^{-2}, respectively.

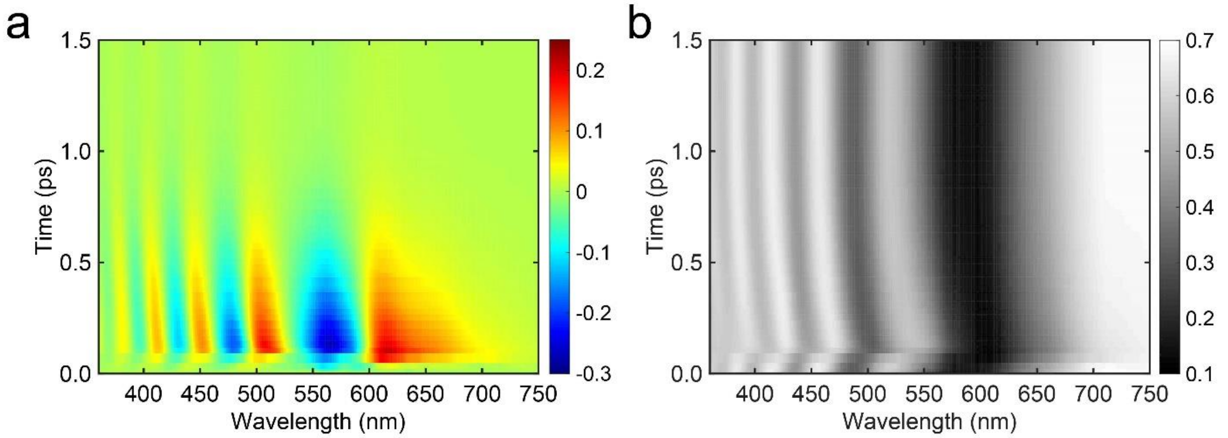

Supplementary Figure 15 | Spectral maps of the change of optical density and total transmission. **a**, **b**, $\Delta OD(t)$ and $T(t)$ spectral maps for the first 1.5 ps under a pump fluence of 3.71 $mJ\cdot cm^{-2}$, which are to be compared with the $\Delta T(t)/T(0)$ spectral map shown in Fig. 2a.

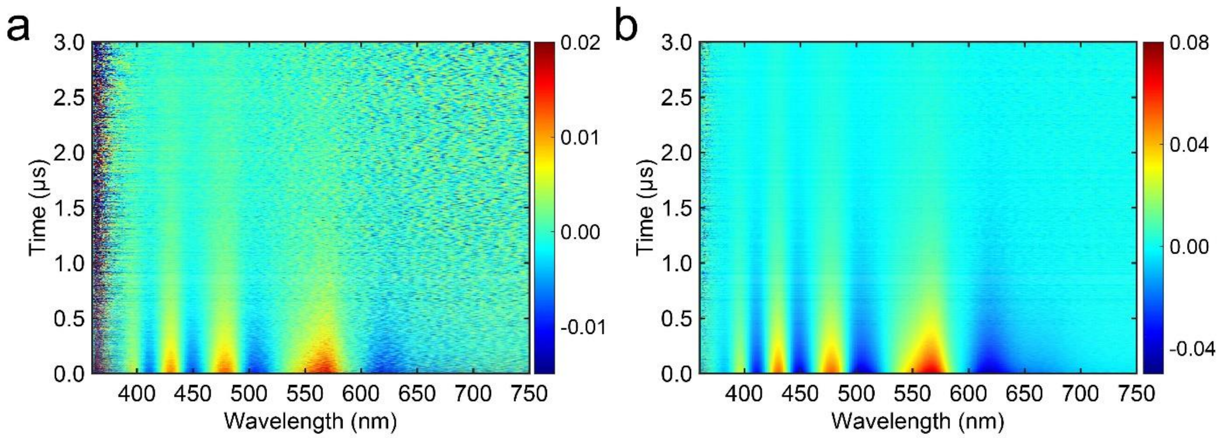

Supplementary Figure 16 | Transient spectral maps of the slow component. $\Delta T(t)/T(0)$ spectral maps plotted for delay times up to 3 μs (acquired from long-delay-TA experiments). Pump fluences for **a** and **b** are 1.31 mJ·cm^{-2} and 9.2 mJ·cm^{-2}, respectively.

Supplementary Note 1: Calculation of the grating order intensities

The electromagnetic waves scattered by a periodic phased array can be decomposed into orthogonal eigenmodes, which are essentially the grating orders including both propagating and evanescent ones. Since the nanorod spacing of 1 μm is comparable to the wavelength in the visible range, higher order propagating modes (besides the zero order mode) can be produced. To extract intensities of these higher order modes from optical simulations, we decomposed the transmitted electric fields according to the procedures shown by J. Jin et al[2]. Briefly, a two dimensional Fourier transform was performed on the electric field at the bottom boundary of the YSZ interface (the array being in the *x*-*y* plane and the bottom boundary is at $z = z_0$),

$$\mathbf{E}(x, y, z_0) = \sum_{n=-\infty}^{+\infty} \sum_{m=-\infty}^{+\infty} \mathbf{E}_{nm}(z_0) \mathbf{exp}\left(-i\left(k_{xn}x + k_{ym}y\right)\right),$$

where the coefficients

$$\mathbf{E}_{nm}(z_0) = \frac{1}{S} \iint \mathbf{E}(x, y, z_0) \mathbf{exp}\left(i\left(k_{xn}x + k_{ym}y\right)\right)$$

are the electric field intensities for the (*n*, *m*) order. $S = a^2$ is the cross-sectional area of a unit cell, $k_{xn} = k_{x0} - 2\pi n/a$ and $k_{ym} = k_{y0} - 2\pi m/a$. Here $k_{x0} = k_0 \cdot \sin\theta \cdot \cos\varphi$, $k_{y0} = k_0 \cdot \sin\theta \cdot \sin\varphi$, $k_0 = 2\pi/\lambda$ is the incident wave vector, $\theta = 0$ is the incident angle and $\varphi = 0$ is the azimuthal angle (the incident wave vector is normal to the substrate). The wave vector in the *z* direction is $k_{znm} = (k_0^2 - k_{xn}^2 - k_{ym}^2)^{1/2}$; a mode is propagating when k_{znm} is real and evanescent when k_{znm} is imaginary.

Transmission of the (*n*, *m*) grating order is calculated as $T_{nm} = \frac{\left|\mathbf{E}_{nm}(z_0)\right|^2}{\left|\mathbf{E}_{inc}\right|^2}$, where $\mathbf{E}_{\text{inc}}$ is the electric field of the incident wave. The (1, 0) and (1, 1) orders are illustrated in the photograph of Supplementary Fig. 1a.

To further verify that the wave propagating along the nanorod follows the fundamental HE_{11} mode, we plot in Supplementary Fig. 1b to Fig. 1f the distributions of the electric field intensity averaged along the length of the nanorod at the wavelengths of the five transmission minima, which are similar to those reported for the HE_{11} mode elsewhere[3, 4], thereby justifying our use of the effective mode index for the estimation of the spectral locations of the transmission minima.

Supplementary Note 2: Permittivity of ITO

The static transmission spectrum of the ITO-NRA from 360 nm to 710 nm was fitted using the Drude-Lorentz model, $\varepsilon(\omega) = \varepsilon_\infty + A_L/(\omega_L^2-\omega^2-i\gamma_L\omega) - \omega_p^2/(\omega^2+i\gamma_p\omega)$. Parameters that yield a good match between the simulated and the experimental transmission spectra are $\varepsilon_\infty = 3.95$, $A_L = (1.4\text{ eV})^2$, $\omega_L = 3.8$ eV, $\gamma_L = 0.01$ eV, $\omega_p = 2.18$ eV, and $\gamma_p = 0.12$ eV. The Drude-Lorentz model was adopted simply to provide reasonable wavelength dependent permittivity for the subsequent waveguide simulations, from which the effective mode index $n_{eff}(\omega)$ can be obtained. The single Lorentz pole is not expected to accurately describe the permittivity of ITO in the ultraviolet range (below 360 nm). In addition, the value of $\omega_p = 2.18$ eV obtained by fitting the visible spectrum is slightly larger than $\omega_p = 2.02$ eV obtained by fitting the NIR spectrum using a pure Drude model described previously[5]; in this work $\omega_p = 2.02$ eV was used for the calculation of the fluence dependent electron distribution. The relative permittivity of ITO in the visible (with the Drude-Lorentz model) and near-infrared range (with the Drude model) are plotted in Supplementary Fig. 2.

The transmission spectrum of a bare YSZ substrate shown in Supplementary Fig. 3a reveals that although the substrate becomes slightly absorptive at wavelengths below 400 nm, its transmission is almost constant from 360 nm to 710 nm. As a result, in the optical simulations we treated the substrate as a lossless dielectric with a constant refractive index[6] of 2.2. In Supplementary Fig. 3b we plot the simulated transmission spectrum of the array, which compares well with the experimental counterpart. The edge length, height and periodicity are 180 nm, 2.6 μm and 1 μm, respectively.

Supplementary Note 3: Transmission and reflection of the ITO-NRAs measured using an integrating sphere

Supplementary Fig. 4 shows the total transmission and reflection spectra in the visible range for the three ITO-NRA samples investigated in this work measured using an integrating sphere to capture the intensities of the higher diffraction orders. Absorption was deduced by subtracting the total transmission and reflection from unity. In this case the transmission minima are significantly diminished, confirming that the spectral features in the visible range are not due to enhanced absorption, as observed for semiconducting nanowires[7-9].

Supplementary Note 4: Theoretical modelling of the permittivity change of the ITO-NRAs

We first generalize the calculation of the imaginary part of the relative permittivity to cover the case of direct interband optical transition in a semiconductor with a non-parabolic conduction band (CB) and a parabolic valence band (VB). The dispersion relations are $\hbar^2k^2/2m_v = E_v$ for holes in the VB, and $\hbar^2k^2/2m_c = E_c+CE_c^2$ for electrons in the CB; here E_v is the hole energy

referenced to the valence band maximum (VBM), E_c is the electron energy referenced to the conduction band minimum (CBM), m_v is the hole effective mass, m_c is the electron effective mass (at CBM), and C is the non-parabolicity of the CB. Both E_c and E_v are taken as positive.

Supplementary Fig. 6 shows the schematic diagram of the considered optical transition and various quantities defined. We denote $R=m_c/m_v$, and$1/m_r=1/m_v+1/m_c$, where m_r is the reduced effective mass. When an incident photon with energy $\hbar\omega$ is absorbed in a direct, interband optical transition, energy conservation dictates that

$$\hbar\omega = E_g+E_c+E_v \qquad \textbf{(1)}.$$

If we let $k^2 = 2m_c(E_c+CE_c^2)/\hbar^2$, equation **(1)** becomes $\hbar\omega = E_g+E_c+R(E_c+CE_c^2)$. This is a quadratic equation in E_c and can be rewritten as $RCE_c^2+(R+1)E_c+(E_g-\hbar\omega) = 0$, with the solution

$$E_c = \frac{-(R+1)+\sqrt{(R+1)^2-4RC(E_g-\hbar\omega)}}{2RC} \qquad \textbf{(2)}.$$

The derivative of E_c with respective to the photon energy $\hbar\omega$ is given by

$$\frac{d(E_c)}{d(\hbar\omega)} = \frac{1}{\sqrt{(R+1)^2-4RC(E_g-\hbar\omega)}} \qquad \textbf{(3)}.$$

The density-of-states (DOS) for electrons at E_c is $\rho(E_c) = \frac{\sqrt{2m_c(E_c+CE_c^{\ 2})}}{\pi^2\hbar^3} m_c(1+2CE_c)$, or equivalently,

$$\rho(E_c) = \frac{1}{2\pi^2}\left(\frac{2m_c}{\hbar^2}\right)^{\frac{3}{2}}(E_c)^{\frac{1}{2}}(1+CE_c)^{\frac{1}{2}}(1+2CE_c) \qquad \textbf{(4)}.$$

Using $\rho(\hbar\omega)d(\hbar\omega)=\rho(E_c)d(E_c)$, where $\rho(\hbar\omega)$ is the joint-density-of-states (JDOS) for optical transition with photon energy $\hbar\omega$, we get $\rho(\hbar\omega) = [d(E_c)/d(\hbar\omega)]\cdot\rho(E_c)$, which can be calculated numerically using equation **(3)** and **(4)**. The absorption coefficient $\alpha(\omega)$ arising from the considered transition can be written as $\alpha(\omega)=\frac{\pi e^2\hbar}{n'(\omega)\varepsilon_0 c m_0^2}\frac{1}{\hbar\omega}M^2\rho(\hbar\omega)\left[f(E_v)-f(E_c)\right]$, where $n'(\omega)$ is the real part of the refractive index and M is the electric dipole matrix element[10]. For the highly doped materials considered here, $f(E_v) = 1$, hence

$$\alpha(\omega)=\frac{\pi e^2\hbar}{n'(\omega)\varepsilon_0 c m_0^2}\frac{1}{\hbar\omega}M^2\rho(\hbar\omega)\left[1-f(E_c)\right] \qquad \textbf{(5)}.$$

Since $\alpha(\omega) = 2\omega n''(\omega)/c$, we can write

$$\varepsilon''(\omega)=2n'(\omega)n''(\omega)=\frac{cn'(\omega)\alpha(\omega)}{\omega} \qquad \textbf{(6)},$$

where $n''(\omega)$ is the imaginary part of the refractive index. Combining equation **(5)** and **(6)** gives

$$\varepsilon''(\omega)=\frac{\pi e^2}{\varepsilon_0 m_0^2\omega^2}M^2\rho(\hbar\omega)\left[1-f(E_c)\right] \qquad \textbf{(7)},$$

which is a dimensionless quantity. Now the intraband optical pumping in our study gives rise to a redistribution of the electrons in the conduction band, whose temperature T can be calculated based on our earlier study[5]. As the Fermi function term $f(E_c)$ in equation (7) is electron temperature dependent, a temperature dependent $\varepsilon''(\omega)$ can be calculated from

$$\Delta\varepsilon''(\omega,T)=-\frac{\pi e^2}{\varepsilon_0 m_0^2\omega^2}M^2\rho(\hbar\omega)\Delta f(E_c,T),$$

where $\Delta\varepsilon''(\omega,T)=\varepsilon''(\omega,T)-\varepsilon''(\omega,T_0)$ and $\Delta f(E_c,T)=f(E_c,T)-f(E_c,T_0)$ with $T_0=300\,\text{K}$ corresponding to the static case. Knowing $\Delta\varepsilon''(\omega, T)$ we can further obtain $\Delta\varepsilon'(\omega, T)$ using the Kramers-Kronig relation, $\Delta\varepsilon'(\omega,T)=\frac{2}{\pi}\mathbf{P}\int_0^\infty d\omega_0\frac{\omega_0\Delta\varepsilon''(\omega_0,T)}{\omega_0^2-\omega^2}$. The procedure described above was used as a model to theoretically calculate the change of real part of the relative permittivity (shown in Fig. 3f) due to the modification of the interband transition under intraband optical pumping, which is the case of the fast component in our TA experiments. Supplementary Fig. 7 presents the calculated $f(E_c, T)$, $\Delta f(E_c, T)$, $E_c(\hbar\omega)$, and $\Delta f(\hbar\omega, T)$ for electron temperature ranging from 300 K to 14,500 K. The $\Delta f(\hbar\omega, T)$ was introduced in Supplementary Fig. 7d to better illustrate the connection between $\Delta f(E_c, T)$ and $\Delta\varepsilon''(\omega, T)$. Note that at high electron temperatures (> 10,500 K), the electron chemical potential μ falls below the CBM, which is a result of conservation of the electron density.

Supplementary Fig. 8 shows the results for $\rho(\hbar\omega)$, $\rho(\hbar\omega)\cdot\Delta f(\hbar\omega)$, $\Delta\varepsilon''(\omega, T)$, as well as the experimentally determined $\alpha(\omega)$ and theoretically calculated $\alpha(\omega)$ on the basis of a constant matrix element of $(2.8\times10^{-49}\ \text{J}\cdot\text{kg})^{1/2}$. Note that various assumptions and simplifications were made for the theoretical calculations: 1) an experimental value of the effective mass for holes has not been reported. Density-functional-theory results all predict a much flatter VB compared to the CB[11-13], hence we assumed a flat VB; 2) the dispersion of the non-parabolic CB[14] is governed by $m_c = 0.263\ m_0$ and $C = 0.4191\ \text{eV}^{-1}$; 3) the band gap is determined by subtracting the theoretically calculated electron Fermi energy referenced to CBM (~ 1.24 eV) from the experimentally measured absorption onset energy for an epitaxial ITO film sputtered on YSZ (~ 4.0 eV as shown in Supplementary Fig. 8d). As it was not possible to get reliable absorption versus wavelength data for the ITO-NRA (arising from the scattering as well as absorption due

to the substrate, which is evident from Supplementary Fig. 4), we performed ellipsometry measurements (in both the ultraviolet to visible, and near-infrared ranges) on an epitaxial ITO film (135 nm thick) sputtered on YSZ, with the results shown in Supplementary Fig. 9. The experimental $\alpha(\omega)$ curve (Supplementary Fig. 8d) was calculated using the refractive index obtained from the ellipsometric data fitting. The film plasma frequency was determined to be ~ 2.1 eV based on the ellipsometry data in the NIR (Supplementary Fig. 9b); this value is close to the deduced plasma frequency of 2.02 eV for the ITO-NRAs.

Supplementary Note 5: Estimating the electron and lattice temperatures

Measurements of the pump power showed that nearly 50% is transmitted and about 5% is reflected (reflection was referenced to a 200 nm thick gold film) by the ITO-NRA for all fluences used in the short-delay-TA experiments (up to 10.72 mJ·cm^{-2}). We therefore conclude that 45% of the pump energy is absorbed by the ITO-NRA. Assuming a spatially uniform excitation profile, the energy (in mJ) deposited per unit volume of ITO nanorod (in cm^3) per pump pulse can be calculated as $\frac{0.45\times p}{L^2H/a^2}$, where p is the pump fluence in mJ·cm^{-2}, L is the edge length in cm, H is the height in cm, and a is the periodicity in cm of the ITO-NRA. Note that in numerical calculations p was treated as a continuous variable.

The electron temperature at $t_{e,0}$ is denoted as $T_{e,0}$. This was estimated using the procedure described earlier[5]. To assess the lattice temperature (denoted as $T_{l,0}$) achieved at $t_{l,0}$, we used the heat capacity data from E. H. P. Cordfunke et al[1] for In_2O_3 measured for the range from 0 to 1000 K. To convert this data into the required units we used the In_2O_3 molecular weight of 277.64 g·mol^{-1} and a mass density of 7.16×10^3 kg·m^{-3} (calculated from the lattice constant of

cubic ITO, 1.01 nm). For comparison purposes, Cordfunke's heat capacity is equivalent to 2.567×10^{6} J·m^{-3}·K^{-1} at 298 K, which is to be compared with a value of 2.58×10^{6} J·m^{-3}·K^{-1} adopted in the independent work by T. Yagi et al[15].

Supplementary Fig. 10a and Fig. 10b show the calculated dependences of $T_{e,0}$ and $T_{l,0}$ on the pump fluence (shown as curves). Based on the experimental fluences we can estimate temperatures reached in the TA experiments (shown as circles). Notably, the initial temperature of electrons (maximum is ~ 14,000 K) is about two orders of magnitude higher than that of the lattice (maximum is ~ 500 K), which stems from their very different heat capacities, as shown in Supplementary Fig. 10c and Fig. 10d.

Supplementary Note 6: Details of the heat-transfer simulations

The heat transfer equation is given by $\rho C_p(\partial T_L/\partial t) + \nabla\cdot(-\kappa\nabla T_L) = 0$ where the temperature T_L is a function of both time and position, and κ is the thermal conductivity. This equation was solved using COMSOL Multiphysics in the time domain. A uniform temperature profile in the nanorod was used as the initial condition (with temperatures obtained from Supplementary Fig. 10b). Periodic boundary conditions were used along the in-plane directions. As no perfectly matched layer (which is an absorbing boundary) is available in the time domain study, we truncated the YSZ substrate in the out-of-plane direction at 10 µm below the interface of YSZ and ITO. A constant temperature (300 K) was imposed on the bottom YSZ boundary; this is valid since the total volume of YSZ is more than two orders of magnitude larger than the nanorod in the heat-transfer simulations, therefore temperature rise at the bottom boundary is at most a few degrees.

The thermal conductivity κ of ITO was calculated from the equation $\kappa = \kappa_{el} + \kappa_{ph}$, where κ_{el} and κ_{ph} are thermal conductivities contributed by mobile electrons and phonons, respectively. According to T. Ashida et al[16], κ_{ph} is almost constant (3.95 $W \cdot m^{-1} \cdot K^{-1}$ for ITO films with different electron concentrations), whereas κ_{el} is well described by the Wiedemann-Franz law of $\kappa_{el} = LT\sigma$, where L is the Lorentz number (2.45×10^{-8} $W\Omega \cdot K^{-2}$) and σ is the electrical conductivity. In our heat-transfer simulations we considered $\kappa_{el} = LT\sigma$ as a temperature dependent quantity, as opposed to κ_{ph} which was assumed to be temperature independent. To get a reasonable estimate for σ, we performed Hall measurement (Van der Pauw method, Ecopia HMS-5000) on an epitaxial ITO film grown on YSZ substrate, whose electron concentration and mobility were found to be ~ 1.3×10^{21} cm^{-3} and 47 $cm^{2} \cdot V^{-1} \cdot s^{-1}$, respectively, yielding a value of 9.4881×10^{5} $S \cdot m^{-1}$ for σ. The thermal conductivity of ITO at 300 K is determined to be 10.9 $W \cdot m^{-1} \cdot K^{-1}$, which is more than an order of magnitude smaller than that of gold (314 $W \cdot m^{-1} \cdot K^{-1}$). The thermal conductivity and heat capacity of YSZ were taken to be 2.5 $W \cdot m^{-1} \cdot K^{-1}$ (from K. W. Schlichting et al[17]) and 60.4 $J \cdot mol^{-1} \cdot K^{-1}$ (from T. Tojo et al[18]), respectively. Both quantities were assumed to be temperature independent, since the temperature rise in the YSZ substrate is negligible in comparison to that of ITO. YSZ's molecular weight and mass density were 123.218 $g \cdot mol^{-1}$ and 6.0 $g \cdot cm^{-3}$, respectively[17]. To further explore the geometrical dependence of the lattice heat dissipation rate, we performed extra heat-transfer simulations for ITO nanorods with different heights and edge lengths; the results are summarized in Supplementary Fig. 12. Interestingly, by adjusting the nanorod height the decay rate can be tuned over an order of magnitude. In contrast, changing the nanorod edge length has a negligible influence on the heat dissipation rate.

Supplementary Note 7: Full $\Delta T(t)/T(0)$ spectral maps

Supplementary Fig. 14 shows the $\Delta T(t)/T(0)$ spectral maps of the fast component acquired from short-delay TA experiments. The transient spectra of $\Delta T(t)/T(0)$ plotted in Fig. 2b are line-cuts from these maps at time delay time $t_{e,0}$. Supplementary Fig. 15 presents the $\Delta T(t)/T(0)$ spectral maps of the slow component obtained from the long-delay TA experiments; the kinetics shown in Fig. 5b are line-cuts from these maps at 560 nm. A low signal-to-noise ratio below 400 nm arises from a relatively weak probe intensity.

Supplementary Note 8: Spectral maps of $\Delta OD(t)$ and $T(t)$

In TA experiments $\frac{\Delta T(t)}{T(0)} = \frac{T(t) - T(0)}{T(0)} = \frac{I(t)/I_0 - I(0)/I_0}{I(0)/I_0}$, where I_0 is the intensity of the beam transmitting through air (taken as the background in all measurements), and $I(0)$ and I(t) are beam intensities transmitting through the sample before and at delay time t after the pump, respectively. Another commonly used quantity, $\Delta OD(t)$, is related to $\Delta T(t)/T(0)$ as, $\Delta OD(t) = -\log_{10}[1+\Delta T(t)/T(0)]$. The $\Delta OD(t)$ spectral map is plotted in Supplementary Fig. 15a for comparison with the corresponding $\Delta T(t)/T(0)$ spectral map (Fig. 2a). In addition, from $T(0)$ and $\Delta T(t)/T(0)$ we can calculate $T(t)$, which is a direct way to present the dynamic transmission property of the array. In Supplementary Fig. 15b we plot $T(t)$ to demonstrate that the visible transmission spectrum first redshifts and then recovers rapidly in sub-picosecond time scales.

References

1. Cordfunke, E.H.P. & Westrum, E.F. The heat capacity and derived thermophysical properties of In_2O3 from 0 to 1000 K. *J. Phys. Chem. Solids* **53**, 361-365 (1992).
2. Jin, J.-M. & Riley, D.J. Finite element analysis of antennas and arrays, Edn. 2nd. (John Wiley & Sons, New Jersey; 2008).
3. van Vugt, L.K., Piccione, B., Cho, C.-H., Nukala, P. & Agarwal, R. One-dimensional polaritons with size-tunable and enhanced coupling strengths in semiconductor nanowires. *Proc. Natl. Acad. Sci.* **108**, 10050-10055 (2011).
4. Brongersma, M.L., Cui, Y. & Fan, S. Light management for photovoltaics using high-index nanostructures. *Nat. Mater.* **13**, 451-460 (2014).
5. Guo, P., Schaller, R.D., Ketterson, J.B. & Chang, R.P.H. Ultrafast switching of tunable infrared plasmons in indium tin oxide nanorod arrays with large absolute amplitude. *Nat. Photonics* **10**, 267-273 (2016).
6. Wood, D.L. & Nassau, K. Refractive index of cubic zirconia stabilized with yttria. *Appl. Opt.* **21**, 2978-2981 (1982).
7. Seo, K. et al. Multicolored vertical silicon nanowires. *Nano Lett.* **11**, 1851-1856 (2011).
8. Cao, L. et al. Engineering light absorption in semiconductor nanowire devices. *Nat. Mater.* **8**, 643-647 (2009).
9. Wang, B. & Leu, P.W. Tunable and selective resonant absorption in vertical nanowires. *Opt. Lett.* **37**, 3756-3758 (2012).
10. Coldren, L.A., Corzine, S.W. & Mašanović, M.L. Diode lasers and photonic integrated circuits. (John Wiley & Sons, Hoboken, New Jersey; 2012).

11. Mryasov, O.N. & Freeman, A.J. Electronic band structure of indium tin oxide and criteria for transparent conducting behavior. *Phys. Rev. B* **64**, 233111 (2001).

12. Oliver, B. Indium oxide—a transparent, wide-band gap semiconductor for (opto)electronic applications. *Semiconductor Science and Technology* **30**, 024001 (2015).

13. Medvedeva, J.E. & Hettiarachchi, C.L. Tuning the properties of complex transparent conducting oxides: Role of crystal symmetry, chemical composition, and carrier generation. *Phys. Rev. B* **81**, 125116 (2010).

14. Liu, X. et al. Quantification and impact of nonparabolicity of the conduction band of indium tin oxide on its plasmonic properties. *Appl. Phys. Lett.* **105**, 181117 (2014).

15. Yagi, T. et al. Analysis on thermal properties of tin doped indium oxide films by picosecond thermoreflectance measurement. *J. Vac. Sci. Technol. A* **23**, 1180-1186 (2005).

16. Ashida, T. et al. Thermal transport properties of polycrystalline tin-doped indium oxide films. *J. Appl. Phys.* **105**, 073709 (2009).

17. Schlichting, K.W., Padture, N.P. & Klemens, P.G. Thermal conductivity of dense and porous yttria-stabilized zirconia. *J. Mater. Sci.* **36**, 3003-3010 (2001).

18. Tojo, T., Atake, T., Mori, T. & Yamamura, H. Heat capacity and thermodynamic functions of zirconia and yttria-stabilized zirconia. *J. Chem. Thermodyn.* **31**, 831-845 (1999).
